# Cationic Nanoparticles Assembled from Natural-Based Steroid Lipid for Improved Intracellular Transport of siRNA and pDNA

**DOI:** 10.3390/nano6040069

**Published:** 2016-04-13

**Authors:** Ruilong Sheng, Xiaoqing Zhuang, Zhao Wang, Amin Cao, Kaili Lin, Julian X. X. Zhu

**Affiliations:** 1 CAS Key Laboratory for Organic Functional Materials, Shanghai Institute of Organic Chemistry, Chinese Academy of Sciences, 345 Lingling Road, Shanghai 200032, China; wangz@mail.sioc.ac.cn; 2 Department of Chemistry, Université de Montréal, C.P.6128, Succursale Centre-ville, Montréal, QC H3C3J7, Canada; julian.zhu@umontreal.ca; 3 General Hospital of Ningxia Medical University, Yinchuan 750004, China; zhuangxiaoqing@gmail.com; 4 School & Hospital of Stomatology, Tongji University, Shanghai Engineering Research Center of Tooth Restoration and Regeneration, 399 Middle Yanchang Road, Shanghai 200072, China

**Keywords:** diosgenin, steroid, siRNA, pDNA, intracellular uptake

## Abstract

Developing new functional biomaterials from biocompatible natural-based resources for gene/drug delivery has attracted increasing attention in recent years. In this work, we prepared a series of cationic nanoparticles (Diosarg-DOPE NPs) by assembly of a natural steroid diosgenin-based cationic lipid (Diosarg) with commercially-available helper lipid 1,2-dioleoyl-sn-glycero-3-phosphorethanolamine (DOPE). These cationic Diosarg-DOPE NPs were able to efficiently bind siRNA and plasmid DNA (pDNA) via electrostatic interactions to form stable, nano-sized cationic lipid nanoparticles instead of lamellar vesicles in aqueous solution. The average particle size, zeta potentials and morphologies of the siRNA and pDNA complexes of the Diosarg-DOPE NPs were examined. The *in vitro* cytotoxicity of NPs depends on the dose and assembly ratio of the Diosarg and DOPE. Notably, the intracellular transportation efficacy of the exogenesis siRNA and pDNA could be greatly improved by using the Diosarg-DOPE NPs as the cargoes in H1299 cell line. The results demonstrated that the self-assembled Diosarg-DOPE NPs could achieve much higher intracellular transport efficiency for siRNA or pDNA than the cationic lipid Diosarg, indicating that the synergetic effect of different functional lipid components may benefit the development of high efficiency nano-scaled gene carriers. Moreover, it could be noted that the traditional “lysosome localization” involved in the intracellular trafficking of the Diosarg and Diosarg-DOPE NPs, indicating the co-assembly of helper lipid DOPE, might not significantly affect the intracellular localization features of the cationic lipids.

## 1. Introduction

In the past few decades, developing functional drug/gene delivery systems derived from natural resources with good biocompatibility, controllable and highly efficient delivery capability, as well as low economic cost has attracted great attention [1,2]. By now, many natural-based hydrophobic blocks (phospholipids, lipoid, steroids, *etc.*) [3] and amine-containing cationic groups [4] (amino acids, polyamine, *etc.*) have been widely employed to construct new biocompatible cationic lipids with the merits of a well-defined and tunable molecular structure [5]. Yang *et al.* [6] developed 1,12-diaminododecane-polyamine cationic bolaamphiphiles with high gene transfection efficiency. Yu *et al.* [7] synthesized some natural lipid-based cyclic polyamines as efficient gene carriers. In a previous study, we designed and synthesized a series of steroid-based cationic lipids, including cholesterol-based bioreduction-responsive cationic lipids (CHOSS) [8], “click” synthesized cholesterol and lithocholate-derived cationic lipids [9], as well as cholesterol-based cationic lipids with versatile amino acid headgroups and chemical linkage bonds [10]. Furthermore, the relationship between their structures and transfection activity had also been preliminarily investigated. Up to date, although many natural products-derived cationic lipids have been synthesized for gene delivery, their self-assembly properties, intracellular uptake, gene transfection efficacy, *etc.*, need to be further controlled and optimized [11]. It is noteworthy for most of the lipid-based gene delivery nano-systems that the intracellular uptake and gene delivery efficiency were still far below their natural virus counterparts. Thereby, optimization of the cationic lipid by covalent and non-covalent approaches is essential for achieving high intracellular transport and efficient gene delivery capability, which needs to be continually investigated.

It had been revealed that the non-covalent binding of functional building blocks by using controlled self-assembly methods could generate new supramolecular aggregates with various structures and functions, which provided possible approaches for optimizing the cationic lipid gene carriers. In recent years, some lipid components, called “helper lipids”, had been employed for non-covalent optimization of the cationic lipids towards highly efficient gene delivery. Up to now, several electrically neutral lipids, including cholesterol [12], phosphatidylcholine (PC) [13], dioleoylphosphatidyl ethanolamine (DOPE) [14], alkylacyl phosphatidylcholines (APC) [15], *etc*., had been utilized as the helper lipids for the preparation of new multi-component cationic lipid formulations as gene delivery carriers. Previous research had revealed that the addition/assembly of helper lipids could bring new functions, such as stabilizing the gene/cationic lipid payload, improving the cellular uptake, facilitating the cell-penetrating ability, and so on [16]. Noteworthy, the co-assembly of cationic lipids with helper lipids could lead to the formation of nanostructures with a controllable and tunable size, shape and morphology [17]. In some cases, the co-assembly of helper lipids could facilitate the formation of nanomicelles/nanoparticles [13,17,18], whereas in some other cases, the introduction of helper lipids could lead to forming fluidic, highly ordered, lamellar-phased nanovesicles/liposomes [19,20,21]. Moreover, the assembled nano-scale structures could convert from one to another under certain conditions. Sakurai *et al.* [22] disclosed a pH-induced structure changing from a micelle to a hexagonally-packed cylinder in a co-assembled DA-DOPE (DA: *N*-(3,5-dialkylbenzyl) ethane-1,2-diamine) lipid system. It has been known that the lipid component, size, shape, morphology and surface charge of assembled cationic nano-aggregates would greatly affect the related gene transfection efficacy, cellular uptake and intracellular distribution of their gene payload. Therefore, the rational design and assembly of the cationic lipids with helper lipids to obtain a controllable, ordered and functionalized lipid formulation as gene carriers with enhanced intracellular gene transport/transfection performances have been regarded as essential issues and challenges in developing the lipid-based gene carriers.

On the other hand, the gene substances pDNA and siRNA both possess negatively-charged phosphodiester skeletons, which make them able to interact with positively-charged cationic lipids (or liposomes and lipid nanoparticles) to form gene-loaded, condensed, nano-scale (100–400 nm) complexes for gene delivery [23]. However, pDNA and siRNA have different molecular weights, scales and topographies. Generally, pDNA are double-stranded, high molecular weight (more than several hundreds of base pairs) biomacromolecules with coiled and supercoiled topostructures, while siRNA are also double-stranded biomacromolecules, but with a much lower molecular weight (normally 21−23 base pairs in length) and with a “rigid-rod” molecular topology [24]. Their difference in physico-chemical properties may offer them different self-assembly capabilities. Consequently, whether a natural lipid-based gene delivery system is suitable for both pDNA and siRNA transportation is still not clear. Moreover, the comparison of the physico-chemical features, cellular uptake, intracellular transport and distribution of the natural lipid-based nano-systems serving as a pDNA and siRNA dual-functional payload was also scarcely reported in earlier literature [25].

**Scheme 1:**
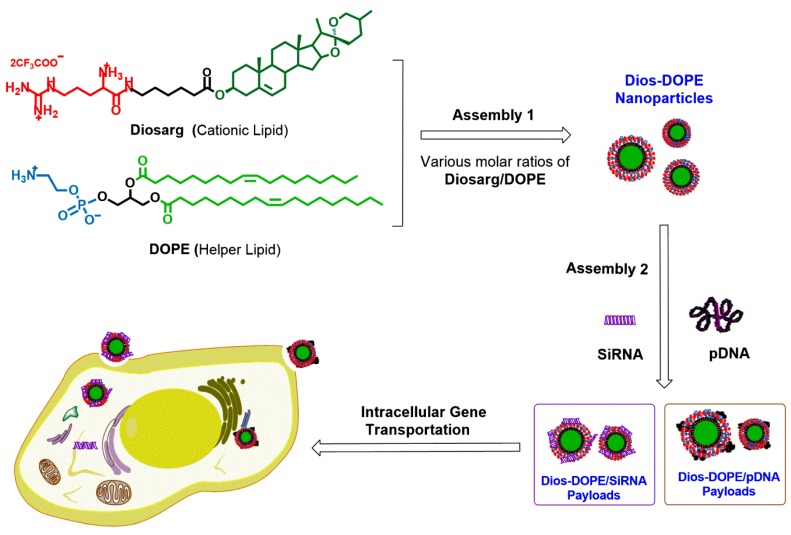
Preparation of Diosarg-1,2-dioleoyl-sn-glycero-3-phosphor-ethanolamine (DOPE) nanoparticles as gene (siRNA and DNA) carriers for intracellular gene transportation.

In this work, we synthesized a natural steroid diosgenin-based cationic lipid (Diosarg) with an arginine-bearing headgroup, which was then co-assembled with a membrane-fusion lipid DOPE (in various molar ratio) to prepare cationic Diosarg-DOPE nanoparticles (NPs). Then, the Diosarg- DOPE NPs were employed as templates for the preparation of pDNA and siRNA payloads (Scheme 1). The physico-chemical properties of the as-prepared cationic lipid/NPs were studied by dynamic light scattering (DLS) instruments and transmission electron microscopy (TEM), and the gene substances’ (pDNA and siRNA) binding affinity, particle size, zeta potential, as well as the morphology of the pDNA/siRNA payloads were studied by DLS, TEM and the agarose-gel retardation assay. The cytotoxicity and gene transfection/transportation properties of the Diosarg lipid and Diosarg-DOPE NPs were measured. In addition, the cellular uptake and intracellular localization of the pDNA/siRNA payloads were also directly observed by flow cytometry (FACS) and fluorescence microscopy, respectively.

## 2. Experimental

### 2.1. Materials

The cationic lipid Diosarg was prepared according to our recent work, and the related chemical synthesis routes and structural characterization are described in detail in the Supplementary Materials (Section S1). 1,2-dioleoyl-sn-glycero-3-phosphor-ethanolamine (DOPE) was purchased from Sigma and Aldrich (St. Louis, MO, USA) was utilized as-received. Luciferase-encoded plasmid DNA [26] (pLuc DNA, 1.0 mg/mL) and human non-small lung carcinoma (H1299) cells were generously gifted by Prof. Yuhong Xu of the School of Pharmacy. Bcl-2 siRNA (Cy3-labeled) was purchased from Sangon Shanghai Co. Ltd (Shanghai, China). Ninety-six-well microplates and 50-mL cell cultivation flasks were purchased from Corning Co. Ltd (Corning, NY, USA). The 0.1 M phosphate buffer solution (PBS), Dulbecco’s Modified Eagle Media (DMEM) and fetal bovine serum (FBS) were supplied by Hangzhou Genom Co. Ltd (Hangzhou, China). The luciferase assay and bicinchoninic acid (BCA) protein quantitation kits were purchased from Promega (Madison, WI, USA) and Biomiga (San Diego, CA, USA), respectively. The label IT tracker™ intracellular nucleic acid localization kit was bought from Mirus Bio Corporation (Madison, WI, USA). In the current study, all of the other chemical reagents and organic solvents were analytical grade and were utilized without further purification.

### 2.2. Analytical Methods

#### 2.2.1. ^1^H NMR Measurements

^1^H NMR spectra were measured and recorded at ambient temperature on a Varian VXR 300 FT-NMR Fourier transform NMR spectrometer instrument (Palo Alto, CA, USA), operating at 300.0 MHz for the ^1^H nuclei. ^13^C NMR spectra were characterized under room temperature on a BrukerAvance 300 NMR spectrometer (Billerica, MA, USA), operating at 75.0 MHz for the ^13^C nuclei, and tetramethylsilane (TMS) was applied as the internal chemical shift reference. Mass spectra (ESI-MS) were measured on a Varian SATURN 2000 spectrometer (Palo Alto, CA, USA).

#### 2.2.2. Preparation of the Self-Assembled Diosarg-DOPE NPs

The as-synthesized cationic lipid Diosarg and helper lipid DOPE [27] were dissolved into a mixture solution of CHCl_3_/CH_3_OH (*v*/*v* = 98/2) at predetermined amounts (molar ratios of 2:1, 1:1 and 1:2), followed by evaporation under reduced pressure to remove the organic solvents and to form a thin lipid film; then, the resulting lipid film was hydrated with distilled water in a supersonic water bath for 30 min at room temperature. Finally, the cationic Diosarg-DOPE NPs stock solutions were further diluted in pure water to prepare the solution with the final Diosarg concentration of 1.7 × 10^−3^ M (for all of the Diosarg and Diosarg-DOPE NPs).

#### 2.2.3. Average Particle Sizes and Surface Charges of the Diosarg-DOPE NPs, siRNA and pDNA-Loaded Complexes Measured by the Dynamic Light Scattering Instrument

For the Diosarg-DOPE NPs, the average particle sizes and surface charges of the as-prepared cationic Diosarg-DOPE (2:1, 1:1, 1:2) NP stock solutions (with the Diosarg concentration of 1.7 × 10^−3^ M for all Diosarg-DOPE NPs) were measured on a Malvern Zetasizer Nano ZS90 (Malvern, Worcestershire, UK) dynamic light scattering instrument (λ = 633 nm) at a fixed scattering angle of 90°.

To evaluate the storage stability of the Diosarg-DOPE NPs, the as-prepared cationic Diosarg-DOPE (2:1, 1:1, 1:2) NP stock solutions were kept at room temperature for a pre-set time (0, 7 and 14 days), then the average particle sizes were measured on a Malvern Zetasizer Nano ZS90 (Malvern, Worcestershire, UK) dynamic light scattering instrument (λ = 633 nm) at a fixed scattering angle of 90°.

For the siRNA and pDNA-loaded complexes, the samples of Diosarg lipid and Diosarg-DOPE NPs were preliminarily dissolved in pure water to prepare the solution with a Diosarg concentration of 1.7 × 10^−3^ M; then, each sample was respectively mixed with Bcl-2 siRNA (5.0 μg) in 1 mL water and pLuc DNA (5.0 μg) in 1 mL water under predetermined +/− charge ratios; then, the mixtures were kept standing within an incubator at 37 °C for 10 min. The average particle sizes and surface charges of the as-prepared Diosarg-DOPE NPs, the Diosarg/siRNA, Diosarg-DOPE NPs/siRNA, Diosarg/pDNA and Diosarg-DOPE NPs/pDNA complexes in aqueous solution were measured on a Malvern Zetasizer Nano ZS90 (Malvern, Worcestershire, UK) dynamic light scattering instrument (λ = 633 nm) at a fixed scattering angle of 90°.

#### 2.2.4. Morphologies of the Diosarg-DOPE NPs and the siRNA and pDNA-Loaded Complexes Measured by Transmission Electronic Microscopy

Each as-prepared solution of the Diosarg-DOPE (2:1, 1:1, 1:2) NPs, Diosarg-DOPE (2:1)/siRNA, Diosarg-DOPE (1:1)/siRNA, Diosarg-DOPE (2:1)/pDNA and Diosarg-DOPE (1:1)/pDNA complexes (+/− = 10) was separately dropped onto a 300-mesh carbon-coated copper grid and air dried at room temperature, then their morphologies under dried conditions were observed and recorded by transmission electron microscopy (TEM, JEOL-1230, JEOL Co. Ltd, Tokyo, Japan) with an acceleration voltage of 80 KV.

#### 2.2.5. MTT Cytotoxicity Assay of the Diosarg Lipid and Diosarg-DOPE NPs

MTT assays were conducted to evaluate the cytotoxicities of the Diosarg lipid and Diosarg-DOPE NPs using H1299 cells. Firstly, the cells were seeded into 96-well microplates with 5 × 10^3^ cells per well in RPMI-1640 medium (100 μL, with 10% FBS) and cultivated under 37 °C (5% CO_2_) for 24 h. Subsequently, the medium was replaced with fresh RPMI-1640 (with 10% FBS), and then, the Diosarg lipid and Diosarg-DOPE NPs under various concentrations were individually added into the wells and further incubated for another 24 h. Then, 20 μL of MTT (5.0 mg/mL) were added into each well and left to incubate for 4 h. After removing the medium, DMSO (100 μL/well) was added to dissolve the formed MTT formazan. Finally, the microplates were dealt with gentle shaking for 10 min to dissolve the formazan, each sample was analyzed with six replicates (*n* = 6) on a microplate reader (BioTek, ELX800, Winooski, VT, USA) at λ = 490 (reference wavelength: λ = 630 nm).

#### 2.2.6. Intracellular Transport of the Cy3-siRNA and Cy3-pDNA-Loaded Complexes of the Diosarg and Diosarg-DOPE NPs Measured by Flow Cytometry and Observed by Fluorescence Microscopy

The Cy3-siRNA and/or Cy3-pDNA-loaded complexes were firstly prepared by direct mixing of the Diosarg lipid and Diosarg-DOPE NPs with Cy3-labeled siRNA (Cy3-siRNA) and pDNA (Cy3-pDNA) and incubated at 37 °C for 20 min. H1299 cells were seeded into 24-well microplates (3 × 10^5^ cells/well in 1 mL DMEM medium with 10% FBS) and incubated at 37 °C under 5% CO_2_ for 24 h and further cultivated overnight in RPMI 1640 medium containing 10% FBS; then, the cell culture medium was replaced with fresh serum-free medium and treated with the Cy3-siRNA and/or Cy3-pDNA-loaded complexes and further incubated for 4 h.

For flow cytometry measurement, the H1299 cells were washed with 1×PBS three times and detached by trypsinization. The cells were harvested by centrifugation and resuspended in 200 μL PBS and transferred to a flow cytometer (BD FACSCalibur, San Jose, CA, USA) for the intracellular transportation assay. During the FACS analysis, the H1299 cells were gated by sideward scatter *versus* forward scatter (SSC/FSC) plots, and the Cy3 fluorescence intensities were recorded in the FL2-H channel.

For fluorescence imaging, the H1299 cells incubated with Cy3-siRNA and/or Cy3-pDNA-loaded complexes (4 h) were washed with 1×PBS three times, then the fluorescent images were observed and recorded on the Nikon Ti-S inverted fluorescence microscope (Tokyo, Japan).

#### 2.2.7. The Observed Intracellular Transport of the Cy3-siRNA and Cy3-pDNA-Loaded Complexes of the Diosarg and Diosarg-DOPE NPs

The as-prepared Diosarg/Cy3-pDNA and Diosarg-DOPE NPs/Cy3-pDNA complexes were added into each well and incubated for 24 h, then washed with 1×PBS three times to eliminate the fluorescence background. After that, the cells were stained with DAPI (for nuclei imaging and localization) for 15 min and washed with 1×PBS three times.

#### 2.2.8. Luciferase Gene Transfection for the pLuc DNA-Loaded Complexes of Diosarg and Diosarg-DOPE NPs

H1299 cells were seeded into 24-well microplates (4 × 10^5^ cells/well) and incubated under 37 °C and 5% CO_2_ with DMEM (10% FBS) for 24 h. The pLuc DNA-loaded complexes of Diosarg and Diosarg-DOPE NPs were prepared by mixing the cationic carriers with luciferase-encoded pLuc DNA (1.0 μg/well) in 200 μL FBS-free DMEM medium at a predetermined +/− charge ratio and incubated for 30 min. Then, the prepared complex solutions were added into the 24-well plates and incubated for 4 h. Afterward, the medium was discarded and replaced with 200 μL fresh DMEM medium (with 10% FBS) and further incubated for 20 h. The luciferase expression assays were conducted in accordance with the protocol of Promega Luciferase assay system, and the total luciferase protein was measured with the BCA assay kit (Applygen Technologies Inc., Beijing, China). Finally, the relative light unit per milligram of luciferase protein (RLU/mg) was calculated to evaluate the cell transfection efficacy of the steroid-based cationic lipids (*n* = 3).

#### 2.2.9. Intracellular Co-Localization of the Diosarg/Cy3-pDNA and Diosarg-DOPE NPs/Cy3-pDNA Complexes Observed by Fluorescence Microscopy

H1299 cells were seeded into 24-well microplates (3 × 10^5^ cells/well in 1 mL DMEM medium with 10% FBS) and incubated at 37 °C under 5% CO_2_ for 24 h. The as-prepared Diosarg/Cy3-pDNA and Diosarg-DOPE NPs/Cy3-pDNA complexes were added into each well and incubated for 24 h, then washed with 1×PBS three times to eliminate the fluorescence background. Thereafter, the cells were stained with DAPI (5 mg/mL, 10 μL/well, for cell nuclei imaging) and Lysotracker (1 mg/mL, 2 μL/well, for lysosome imaging) for 20 min and washed with 1×PBS three times. Finally, the fluorescent images were observed and recorded on the Nikon Ti-S invert fluorescence microscope.

## 3. Results and Discussion

### 3.1. Preparation of Diosarg-DOPE Nanoparticles and Their Physico-Chemical Properties

**Figure 1:**
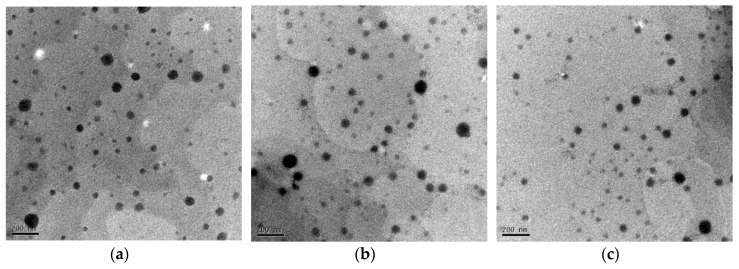
Transmission electron microscopy (TEM) photographs of the Diosarg-DOPE aggregates: (**a**) Diosarg-DOPE (2:1) nanoparticles (NPs); (**b**) Diosarg-DOPE (1:1) NPs; (**c**) Diosarg-DOPE (1:2) NPs; scale bar: 200 nm.

**Figure 2:**
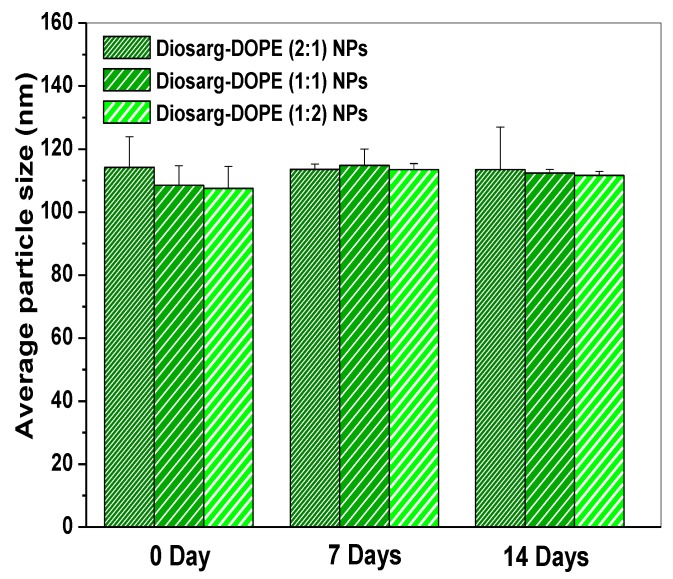
Storage stability of the Diosarg-DOPE (2:1, 1:1, 1:2) NPs in distilled water after 0 day, 7 days and 14 days; the average particle size was measured by the dynamic light scattering instrument.

The cationic lipid Diosarg was synthesized and characterized according to our current work (unpublished) and utilized as a control (Section S1 and Figure S1, Supplementary Materials). Then, the synthesized Diosarg lipid was co-assembled with various molar ratios of commercially-available helper lipid 1,2-dioleoyl-sn-glycero-3-phosphor-ethanolamine (DOPE) by the thin-film hydration method to formulate the Diosarg-DOPE lipid aggregates [28]. The average size, surface potential and morphology of the as-prepared Diosarg-DOPE aggregates were individually characterized by DLS and TEM. As shown in Table 1, no aggregates of the Diosarg lipid itself could be detected by DLS due to its high solubility in aqueous solution. Meanwhile, the Diosarg-DOPE (2:1, 1:1, 1:2) could form nano-scaled aggregates with a hydrodynamic average particle size of 105–117 nm (DLS curves shown in Figure S2, Supplementary Materials) and a zeta potential of 46.8–49.5 mv, and the morphology of these nano-aggregates were further observed as sphere-like nanoparticles (Diosarg-DOPE NPs) with 40–110-nm diameters under TEM (Figure 1), indicating that the addition of the helper lipid DOPE would not significantly change the morphology of the aggregates. Moreover, the comparatively small particle size and positive surface charge might benefit the gene (pDNA, siRNA, *etc*.) loading, endocytosis and intracellular delivery [29]. Moreover, the solution stability was regarded as an important parameter of nano-formulations; the Diosarg-DOPE NPs was put in distilled water at ambient temperature for several days, and their stability was evaluated by determining the particles sizes by DLS at different time points (Figure 2). The results showed that the Diosarg-DOPE NPs could keep the average particle size at relatively stable level (106–116 nm) up to 14 days. The high solution stability was due to the double-chain-bearing DOPE lipid, which enhanced the hydrophobic interactions in the core structure of the Diosarg-DOPE NPs [30]. The high storage stability at room temperature might offer Diosarg-DOPE NPs potential nano-formulations in practical applications.

### 3.2. Average Particle Size, Surface Potential and Morphology of the siRNA- and pDNA-Loaded Complexes

**Figure 3:**
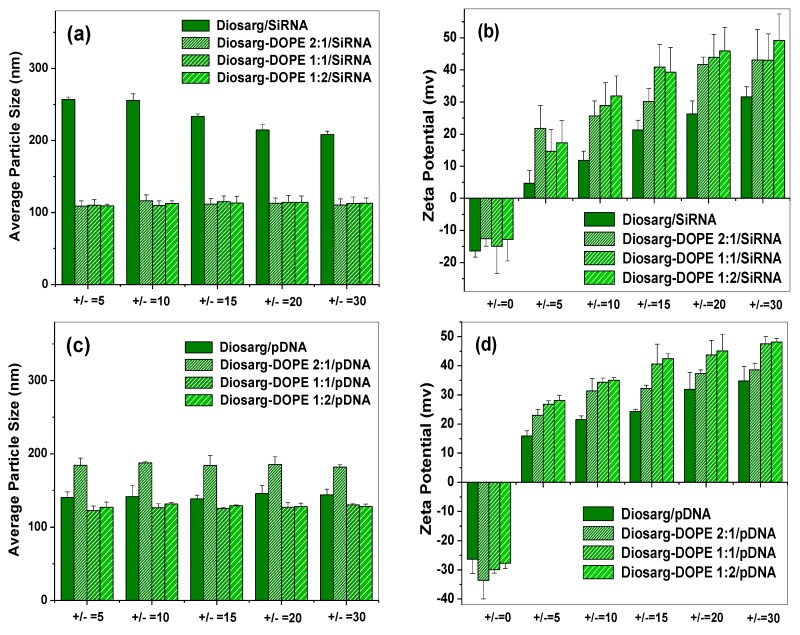
Average particle sizes and zeta potentials of the Diosarg/siRNA and Diosarg-DOPE NPs/siRNA (**a**,**b**), as well as the Diosarg/pDNA (**c**) and Diosarg-DOPE NPs/pDNA (**d**) complexes under the +/− charge ratios from 0 to 30 determined by DLS.

It is known that the average particle size, shape/morphology, surface potential, as well as the particle distribution played essential roles in the related endocytosis and intracellular trafficking/localization [31]. Hereby, to investigate the aggregation properties of the Diosarg and Diosarg-DOPE NPs as the siRNA and pDNA carriers, the average particle size, surface potential and morphology of the Diosarg-DOPE NPs/gene complexes were evaluated by DLS in distilled water. For siRNA-loaded complexes, the Bcl-2 siRNAs were assembled/loaded with Diosarg and Diosarg-DOPE NPs to form the siRNA complexes. As shown in Figure 3a, the Diosarg/siRNA complexes were 210–260 nm diameter aggregates (+/− ratio 5–30), while Diosarg-DOPE (2:1, 1:1, 1:2) NPs/siRNA showed comparably smaller particle sizes at around 112–125 nm, indicating that the co-assembly of DOPE with Diosarg could facilitate the siRNA condensation [32]; in which the Diosarg-DOPE NPs could serve as a template for siRNA assembling/loading. Accordingly, it can be seen in Figure 3b that within the +/− ratio of 10–30, the surface zeta potentials were in the sequence of Diosarg-DOPE (1:2)/siRNA (+32.2–+49.8 mv) > Diosarg-DOPE(1:1)/siRNA (+27.5–+43.8 mv) > Diosarg-DOPE(2:1)/siRNA (+25.1–+43.4 mv) > Diosarg/siRNA (+12.5–+31.6 mv), indicating that the co-assembly of DOPE with Diosarg could lead to the increasing of the surface potentials of the Diosarg-DOPE/siRNA payloads, which might facilitate charge-induced cellular adhesion and intracellular uptake of the assembled Diosarg-DOPE NPs. Similarly, as for pDNA-loaded complexes, the average particle size and surface potential of the Diosarg/pDNA and Diosarg-DOPE NPs/pDNA aggregates were characterized in different +/− charge ratios. As depicted in Figure 3c, Diosarg/pDNA was 131–144 nm-sized aggregates under the +/− charge ratio from 5 to 30; Diosarg-DOPE (2:1)/pDNA was nano-aggregates with 172–188-nm diameters within the same +/− charge ratio. Meanwhile, Diosarg-DOPE (1:1)/pDNA and Diosarg-DOPE (1:2)/pDNA showed comparably smaller particle sizes at about 118–130 nm, indicating that the co-assembly of DOPE could enhance the pDNA condensation effect to some extent [32]. Accordingly, Figure 3d shows that the surface potential converted from negative to positive with the +/− ratio of 0–5 and then gradually increased with the increasing of the +/− ratio from 10 to 30; the surface zeta potentials were in the sequence of Diosarg-DOPE(1:2)/pDNA(+28.2–+49.0 mv) ≥ Diosarg-DOPE(1:1)/pDNA (+24.5–+48.8 mv) > Diosarg-DOPE(2:1)/pDNA (+22.1–+39.4 mv) > Diosarg/pDNA (+15.5–+32.4 mv). Moreover, the pDNA binding affinity of Diosarg and Diosarg-DOPE nanoparticles was further examined by the pDNA retardation assay [31]. Diosarg inhibited the migration of pDNA completely at the +/− charge ratio of 2–4; the Diosarg-DOPE nanoparticles (2:1, 1:1, 1:2) almost have a similar pDNA binding affinity (retarded at +/− 2–4) to that of Diosarg (Figure S3, Supplementary Materials), which is in accordance with the DLS data. Moreover, the results demonstrated the co-assembly of DOPE to Diosarg could lead to the increasing of the surface potentials of the pDNA payloads, which might further improve the pDNA condensing capability of the NPs [33].

Furthermore, we studied the morphology of Diosarg-DOPE/siRNA and Diosarg-DOPE/pDNA complexes (+/− = 15) by TEM. As shown in Figure S4 (Supplementary Materials), similar to the Diosarg-DOPE NPs, the Diosarg-DOPE (2:1)/siRNA and Diosarg-DOPE (1:1)/siRNA complexes were spherical-shaped nanoparticles with an average size of 30–102 nm. The Diosarg-DOPE (2:1)/pDNA and Diosarg-DOPE (1:1)/pDNA complexes were also spherically-shaped nanoparticles with a larger average size of 80–170 nm, and the trend of the TEM is in accordance with the DLS results. It could be noted that all of the siRNA and pDNA-loaded nanoparticles are solid lipid nanoparticles instead of hollow lamella-structured liposome vesicles [34] or tubule-shaped aggregates [35], since the pre-assembled Diosarg-DOPE NPs could serve as nanoparticle templates for the binding/loading of siRNA and/or pDNA in aqueous solution. The previous studies had pointed out that the helper lipid DOPE prefers to aggregate and form non-bilayer lipid aggregate structures with a hexagonal phase (H_II_) instead of a lamellar phase (L_α_) [36]. In addition, the particle size observed by TEM was smaller than that measured by DLS, which could be explained as nanoparticle shrinkage during the drying process in TEM sample preparation [37].

### 3.3. Cytotoxicity of the Diosarg Lipid and Diosarg-DOPE NPs in H1299 Cells by the MTT Assay

**Figure 4:**
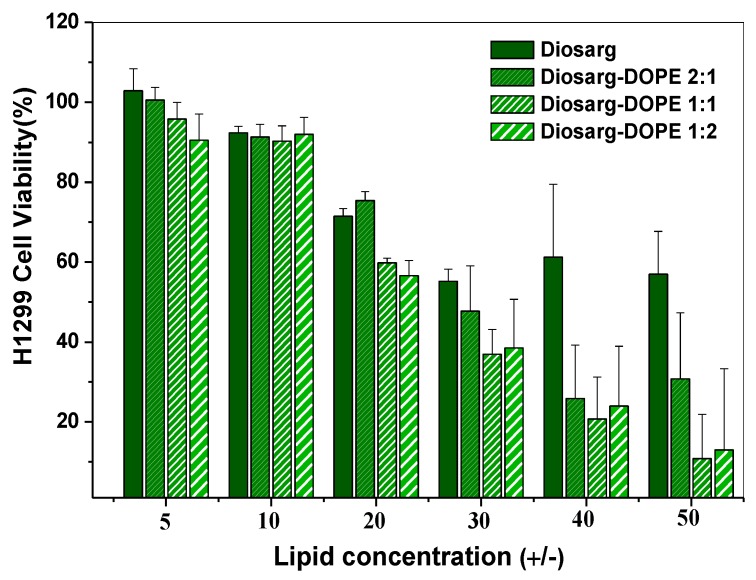
Cytotoxicity of H1299 cells incubated with the Diosarg lipid and Diosarg-DOPE NPs at various +/− charge ratios (pDNA 0.5 μg/well), with the Diosarg lipid at the same +/− charge ratio as the control.

Low cytotoxic and highly biocompatible cationic gene carriers/vectors are an important prerequisite for intracellular gene delivery and clinical gene therapy applications [38]. Hereby, the *in vitro* cytotoxicity of the cationic lipid Diosarg and Diosarg-DOPE NPs was examined by the MTT assay using the H1299 (human lung cancer) cell line. As shown in Figure 4, comparatively high cell viabilities of the Diosarg lipid (>93.0%) and Diosarg-DOPE NPs (>90.1%) were observed at the dose of +/− = 5–10 (the pDNA amount was set as 0.5 μg/well), whereas higher cell viability was observed with the increasing of the +/− charge ratios, due to the membrane disruption induced by the higher positive charge. Noteworthy, the Diosarg-DOPE NPs showed higher cytotoxicity than that of the Diosarg lipids, possibly due to the membrane fusion effect of co-assembled DOPE lipid that caused the decreasing of cell membrane integrity. Moreover, the cytotoxicity was gradually increased with the increasing of the DOPE ratio from 2:1 to 1:2. The results indicated that the cytotoxicity depended largely on the type and proportion of the co-assembled helper lipids. Likewise, Leo *et al.* [39] recently revealed that incorporation of a helper lipid phosphatidylcholine (PC) could bring higher cytotoxicity than the traditional helper lipid of cholesterol. DOPE was known as the main helper lipid component of the commercially-available transfection agent lipofectamine [40], which have relatively high cytotoxicity (especially at higher doses) [41], which may limit practical/clinical application. Thus, rational optimization of the cationic lipids and membrane fusogenic helper lipids toward high biocompatibility and low cytotoxic gene transfection should be further investigated.

### 3.4. Intracellular Transportation of the Cy3-siRNA- and Cy3-DNA-Loaded Complexes Measured by Flow Cytometry and by Fluorescent Imaging

**Figure 5:**
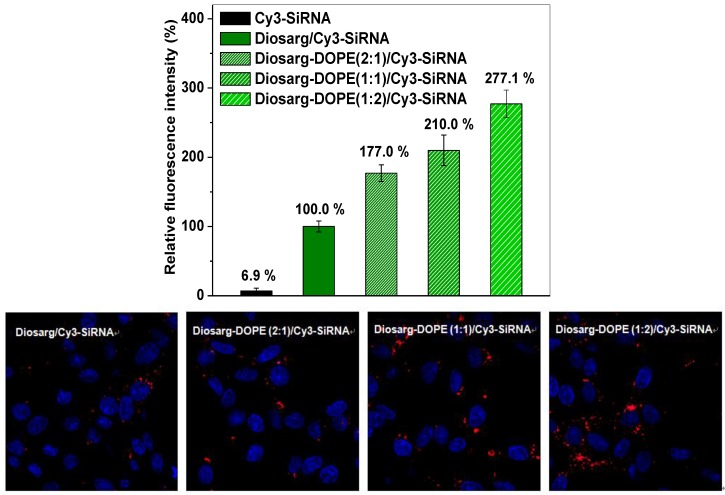
Intracellular uptake of the Diosarg lipid and Diosarg-DOPE (2:1, 1:1, 1:2) NPs/Cy3-siRNA complexes (+/− = 10) in H1299 cells measured by flow cytometry (10,000 cells for each sample) and observed by fluorescence microscopy (blue: DAPI (4’,6-diamidino-2-phenylindole) stained nuclei; red: Cy3-labeled siRNA).

First, the Diosarg/Cy3-siRNA and Diosarg-DOPE NPs/Cy3-siRNA complexes were prepared by direct mixing of the Diosarg lipid and Diosarg-DOPE NPs with Cy3-labeled Bcl-2 siRNA (+/− = 10) [42]; the intracellular transportation of Diosarg/Cy3-siRNA and Diosarg-DOPE/Cy3-siRNA complexes was examined using H1299 cells by flow cytometry. The Cy3 fluorescence intensity of the H1299 cells transfected with the Diosarg-DOPE NPs/Cy3- siRNA complexes was thus calculated as the intracellular transportation efficacy (the related fluorescence intensity of the cells transfected by Diosarg/Cy3-siRNA (+/− = 10) was set as 100%) [8]. The original flow cytometry data are shown in Figure S5 and Figure S6 (Supplementary Materials). As shown in Figure 5, the Diosarg-DOPE (2:1)/Cy3-siRNA, Diosarg-DOPE (1:1)/Cy3-siRNA and Diosarg-DOPE (1:2)/Cy3-siRNA showed relative intracellular uptake capabilities of 1.77-fold (177.0%), 2.10-fold (210.0%) and 2.77-fold (277.1%) higher to that of the control Diosarg/Cy3-siRNA complex (100.0%), respectively. This indicates that the co-assembly of Diosarg with DOPE as the helper lipid could apparently improve the related intracellular transportation capabilities of the nano-scale siRNA payloads. Moreover, the intracellular uptake and localization of the Diosarg lipid and Diosarg-DOPE (2:1, 1:1, 1:2)/Cy3-siRNA complexes were observed under fluorescence microscopy (Figure 5). The results showed that Diosarg and Diosarg-DOPE (2:1)/Cy3-siRNA separately distributed inside the cells, while a relatively large amount of Diosarg-DOPE (1:1 and 1:2)/Cy3-siRNA localized inside the cytoplasm, indicating that the incorporation of helper lipid DOPE was able to improve the intracellular transportation efficacy of the Diosarg-based siRNA payloads.

**Figure 6:**
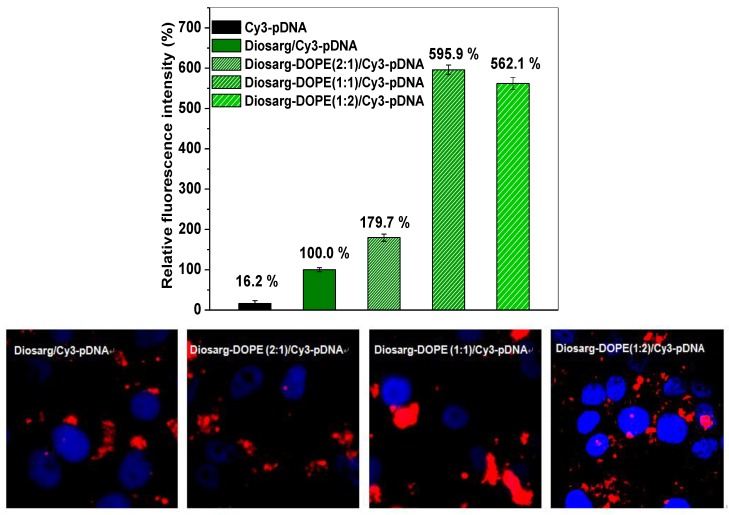
Intracellular uptake of the Diosarg lipid and Diosarg-DOPE (2:1, 1:1, 1:2)/Cy3-pDNA complexes (+/− = 10) in H1299 cells measured by flow cytometry (10,000 cells for each sample) and observed by fluorescence microscopy (blue: DAPI stained nuclei; red: Cy3-labeled pDNA).

Similar to the Cy3-siRNA-loaded complexes, Diosarg/Cy3-pDNA and Diosarg-DOPE NPs/Cy3-pDNA complexes were also prepared by direct mixing of the Diosarg lipid and Diosarg-DOPE NPs with Cy3-labeled pDNA (+/− = 10) [43], respectively. The intracellular transportation efficacy of the Diosarg/Cy3-pDNA and Diosarg-DOPE NPs/Cy3-pDNA complexes was examined in H1299 cells by flow cytometry, and the Diosarg/Cy3-pDNA (+/− = 10) was set as the 100% reference. As shown in Figure 6, the Diosarg-DOPE (2:1) NPs/Cy3-pDNA, Diosarg-DOPE (2:1) NPs/Cy3-pDNA and Diosarg-DOPE (1:2) NPs/Cy3-pDNA showed relative intracellular uptake capabilities of 1.79-fold (179.7%), 5.95-fold (595.9%) and 5.62-fold (562.1%) to that for the Diosarg (100.0%), respectively. The results indicated that the co-assembly of Diosarg with DOPE could largely improve the related intracellular transportation efficacy. Furthermore, the intracellular uptake behaviors of the Diosarg lipid/Cy3-pDNA and Diosarg-DOPE (2:1, 1:1, 1:2)/Cy3-pDNA complexes were observed under fluorescence microscopy (Figure 6 and Figure S7, Supplementary Materials); it could be seen that Diosarg/Cy3-pDNA separately distributed in the H1299 cells. Notably, the Diosarg-DOPE (1:1)/Cy3-pDNA complexes partially localized around and/or inside the cell nuclei. In a prior study, we found that the pDNA-loaded complexes of some cholesterol-based bioreduction-responsive cationic lipids (CHOSS) were able to localize in the perinuclear region, which might benefit the pDNA expression inside the cell nuclei [8]. Thus, we deduced that the perinuclear localization of the Diosarg-DOPE (1:1)/Cy3-pDNA may facilitate the related pDNA expression. Prior research revealed that the headgroup and hydrophobic tail provide a dominant contribution to the cellular fate of the lipid gene carriers [4]. Herein, the results indicated that the amount of the helper lipid DOPE incorporated into the cationic lipid Diosarg gene carriers could largely affect the intracellular localization; it could be expected that the intracellular transportation efficacy might be further optimized by selecting the proper helper lipids as the building blocks for the nano-scale gene/drug delivery systems.

### 3.5. Luciferase Gene Transfection Efficiency of the pLuc DNA-Loaded Complexes

**Figure 7:**
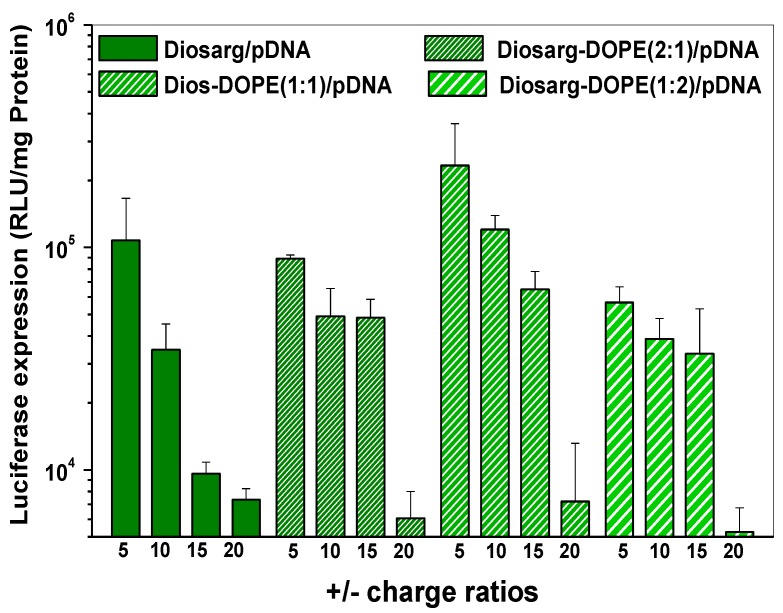
*In vitro* luciferase gene transfection efficiencies of the Diosarg lipid and Diosarg-DOPE (2:1, 1:1, 1:2) NPs/pDNA complexes at various +/− charge ratios in H1299 cells.

Prior works have revealed that the insertion of helper lipids, such as DOPE and DOTAP (1,2-Dioleoyl-3-trimethylammonium-propane chloride), into cationic liposomes could enhance the gene transfection properties, change the related membrane fusion properties, as well as influence the subsequent intracellular gene releasing manners [44,45]. Herein, the luciferase pDNA transfection capabilities of the Diosarg lipid and Diosarg-DOPE (1:1, 1:2, 2:1) NPs were examined by luciferase gene transfection assay. As shown in Figure 7, the Diosarg lipid and Diosarg-DOPE NPs showed different pDNA transfection capacities, Diosarg showed drastic decreasing of the gene transfection efficacy with the increasing of the +/− charge ratios. Diosarg-DOPE (2:1)/pDNA and Diosarg-DOPE (1:2)/pDNA complexes showed comparable slow drop trends in their transfection efficacy within the +/− charge ratio of 5–15, although their transfection efficacy decreased to some extent, due to the template effect of the Diosarg-DOPE NPs. Notably, the Diosarg-DOPE (1:1)/pDNA showed the highest pDNA transfection efficiency among the complexes. Sakurai *et al*. disclosed similar results in cationic lipid DA-DOPE assembled nanoparticles, in which the composition of DA:DOPE = 1:1 showed the highest gene transfection efficiency [22]. The trend of pDNA transfection efficiency resembled the intracellular transportation efficacy (Figure 6), and the Diosarg-DOPE (1:1)/pDNA complexes have the highest intracellular transportation efficacy, which suggested that the intracellular transportation efficacy and subsequent gene delivery capacity could be controlled/optimized by changing the helper lipid ratios.

### 3.6. Intracellular Co-Localization of Diosarg/Cy3-pDNA and Diosarg-DOPE NPs/Cy3-pDNA Complexes by Fluorescent Imaging

**Figure 8:**
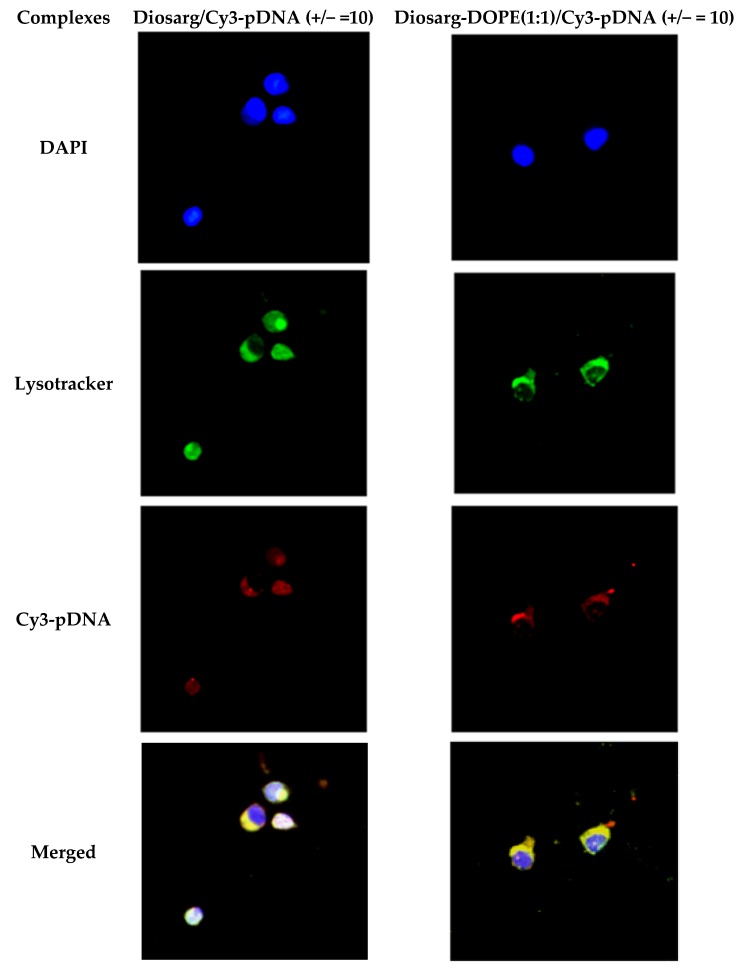
Intracellular localization of the Diosarg lipid and Diosarg-DOPE (1:1)/pDNA complexes (+/− = 10) in H1299 cells observed and recorded by fluorescence microscopy (blue: DAPI stained cell nuclei; green: Lysotracker-stained lysosome; red: Cy3-labeled pDNA).

It had been revealed that the proposed “endocytosis-endosome/lysosome localization-cell nuclei” pathway was recognized as the dominant intracellular trafficking route of many cationic polymer/lipid gene carriers [46,47]. In order to investigate the intracellular localization of Diosarg/Cy3-pDNA and Diosarg-DOPE (1:1)/Cy3-pDNA complexes, the lysosome organelles of H1299 cells were stained with a lysosomal-specific labeling fluorescent agent Lysotracker-green, and the cell nuclei were stained with blue fluorescent DAPI [48]. As shown in Figure 8, after 24 h incubation of fluorescent Diosarg/Cy3-pDNA and Diosarg-DOPE/Cy3-pDNA complexes, the obvious red fluorescence of Cy3-pDNA could be clearly observed in the cytoplasm, while Diosarg-DOPE (1:1)/Cy3-pDNA showed some nucleic localization. Moreover, a large amount of red fluorescent points dispersed inside the cell indicated that both the Diosarg/Cy3-pDNA and Diosarg-DOPE (1:1)/Cy3-pDNA complexes could be effectively uptaken by H1299 cells. Significantly, an obvious co-localization of red fluorescence (Cy3-pDNA) and green fluorescence (Lysotracker) was observed (Figure 8, merged images), indicating that the Diosarg and Diosarg-DOPE NPs complexes both undergo the traditional “lysosomal localization” process. Besides, it could be observed that some red fluorescence points (Cy3-pDNA) partially co-localized with the cell nuclei (blue fluorescence, DAPI), indicating the possible cationic lipid carrier-mediated cross nuclear membrane transportation of Cy3-pDNA. Our previous work has revealed that some natural arginine-rich peptide mimics could result in the nuclear membrane permeation effect [43]; except that some steroid compound, such as cholesterol and glucocorticoids, were able to bind the NF-κB receptor [49], which could accelerate the permeability across the nuclear membrane. The results demonstrated that the intracellular localization would not be significantly affected by co-assembly of DOPE with Diosarg lipid. Moreover, the study of the localization of the steroid-based lipid NPs with other sub-cellular organelles, such as mitochondria, endoplasmic reticulum, as well as the Golgi apparatus [50], has been carried out in our lab.

## 4. Conclusions

In sum, we successfully prepared a series of cationic Diosarg-DOPE NPs self-assembled from a cationic lipid Diosarg and a membrane-fusion lipid DOPE under supersonic hydration conditions. The self-assembled Diosarg-DOPE NPs aggregated into spherical lipid nanoparticles with an average particle size of 105–116 nm, which were able to efficiently bind siRNA and plasmid DNA (pDNA) via electrostatic interactions to form stable, nano-sized cationic lipid nanoparticles instead of lamellar vesicles in aqueous solution. The MTT assay results indicated that the *in vitro* cytotoxicity of the Diosarg-DOPE NPs depended on the dose and assembly ratio of the Diosarg and DOPE. By using the Diosarg-DOPE NPs as the cargoes, the intracellular transportation efficacy of the exogenesis siRNA and pDNA could be greatly improved in H1299 cell line. The improved luciferase expression was also observed by using Diosarg-DOPE NPs as the pDNA carriers. The results demonstrated that the self-assembled Diosarg-DOPE NPs could achieve higher intracellular transport efficiency for siRNA or pDNA than that for the cationic lipid Diosarg, indicating that the non-covalent incorporation of helper lipid DOPE could improve the intracellular gene transportation. The traditional “lysosome localization” involved in the intracellular trafficking of the Diosarg and Diosarg-DOPE NPs indicated that the co-assembly of helper lipid DOPE may not significantly affect the intracellular localization features of the cationic lipids. The results provided deeper understanding of the intracellular uptake and localization manners of self-assembled lipid nanocarriers as efficient intracellular siRNA and pDNA delivery systems. Moreover, the synergistic effect [51] of different functional lipid components may benefit developing two- or multi-component supramolecular systems as highly efficient nano-biomaterials.

## Figures and Tables

**Table 1:** Hydrodynamic average particle size, polydispersity (PDI) and zeta potential of the Diosarg-DOPE aggregates as obtained from the dynamic light scattering (DLS) measurements.

Cationic Carrier Samples	Hydrodynamic Mean Particle Size (nm) ^a^	PDI	Zeta Potential (mv)
Diosarg	/	/	/
Diosarg-DOPE (2:1) NPs	116 ± 2	0.275	49.1 ± 5.1
Diosarg-DOPE (1:1) NPs	117 ± 3	0.225	46.8 ± 7.2
Diosarg-DOPE (1:2) NPs	105 ± 2	0.274	49.5 ± 5.6

^a^ The hydrodynamic average particle size was measured by the DLS instrument at room temperature; the data are presented as the mean ± the standard deviation (SD, *n* = 3).

## Supplementary Materials

The following are available online at http://www.mdpi.com/2079-4991/6/4/69/s1.

## References

[1] Biju V. (2014). Chemical modifications and bioconjugate reactions of nanomaterials for sensing, imaging, drug delivery and therapy. Chem. Soc. Rev..

[2] Fortier C., Durocher Y., De Crescenzo G. (2014). Surface modification of nonviral nanocarriers for enhanced gene delivery. Nanomedicine.

[3] Zhi D., Zhang S., Wang B., Zhao Y., Yang B., Yu S. (2010). Transfection efficiency of cationic lipids with different hydrophobic domains in gene delivery. Bioconjugate Chem..

[4] Zhi D., Zhang S., Cui S., Zhao Y., Wang Y., Zhao D. (2013). The headgroup evolution of cationic lipids for gene delivery. Bioconjugate Chem..

[5] Bhattacharya S., Biswas J. (2009). Understanding membranes through the molecular design of lipids. Langmuir.

[6] Khan M., Ang C.Y., Wiradharma N., Yong L.-K., Liu S., Liu L., Gao S., Yang Y.Y. (2012). Diaminododecane-based cationic bolaamphiphile as a non-viral gene delivery carrier. Biomaterials.

[7] Yi W.J., Zhang Q.F., Zhang J., Liu Q., Ren L., Chen Q.M., Guo L., Yu X.Q. (2014). Cyclen-based lipidic oligomers as potential gene delivery vehicles. Acta Biomater..

[8] Sheng R., Luo T., Zhu Y., Li H., Sun J., Chen S., Sun W., Cao A. (2011). The intracellular plasmid DNA localization of cationic reducible cholesterol-disulfide lipids. Biomaterials.

[9] Sheng R., Luo T., Li H., Sun J., Wang Z., Cao A. (2013). ‘Click’ synthesized sterol-based cationic lipids as gene carriers, and the effect of skeletons and headgroups on gene delivery. Bioorg. Med. Chem..

[10] Sheng R., Luo T., Li H., Sun J., Wang Z., Cao A. (2014). Cholesterol-based cationic lipids for gene delivery: Contribution of molecular structure factors to physico-chemical and biological properties. Colloid. Surf. B.

[11] Zhang X.X., McIntosh T.J., Grinstaff M.W. (2012). Functional lipids and lipoplexes for improved gene delivery. Biochimie.

[12] Sakurai F., Nishioka T., Yamashita F., Takakura Y., Hashida M. (2001). Effects of erythrocytes and serum proteins on lung accumulation of lipoplexes containing cholesterol or DOPE as a helper lipid in the single-pass rat lung perfusion system. Eur. J. Pharm. Biopharm..

[13] Wang X., Yu B., Ren W., Mo X., Zhou C., He H., Jia H., Wang L., Jacob S.T., Lee R.J. (2013). Enhanced hepatic delivery of siRNA and microRNA using oleic acid based lipid nanoparticle formulations. J. Control. Release.

[14] Sun S., Wang M., Alberti K.A., Choy A., Xu Q. (2013). DOPE facilitates quaternized lipidoids (QLDs) for *in vitro* DNA delivery. Nanomedicine.

[15] Huang Z., Li W., Szoka F.C. (2012). Asymmetric 1-alkyl-2-acyl phosphatidylcholine: A helper lipid for enhanced non-viral gene delivery. Int. J. Pharm..

[16] Scarzello M., Chupin V., Wagenaar A., Stuart M.C. A., Engberts J.B.F.N., Hulst R. (2005). Polymorphism of pyridinium amphiphiles for gene delivery: Influence of ionic strength, helper lipid content, and plasmid DNA complexation. Biophys. J..

[17] Tabatt K., Kneuer C., Sameti M., Olbrich C., Müller R.H., Lehr C.M., Bakowsky U. (2004). Transfection with different colloidal systems: Comparison of solid lipid nanoparticles and liposomes. J. Control. Release.

[18] Hattori Y., Nakamura T., Ohno H., Fujii N., Maitani Y. (2013). siRNA delivery into tumor cells by lipid-based nanoparticles composed of hydroxyethylated cholesteryl triamine. Int. J. Pharm..

[19] Felnerova D., Viret J.F., Glück R., Moser C. (2004). Liposomes and virosomes as delivery systems for antigens, nucleic acids and drugs. Curr. Opin. Biotech..

[20] Lonez C., Vandenbranden M., Ruysschaert J.M. (2008). Cationic liposomal lipids: From gene carriers to cell signaling. Prog. Lipid Res..

[21] Ramezani M., Khoshhamdam M., Dehshahri A., Malaekeh-Nikouei B. (2009). The influence of size, lipid composition and bilayer fluidity of cationic liposomes on the transfection efficiency of nanolipoplexes. Colloid. Surf. B.

[22] Mochizuki S., Kanegae N., Nishina K., Kamikawa Y., Koiwai K., Masunaga H., Sakurai K. (2013). The role of the helper lipid dioleoylphosphatidylethanolamine (DOPE) for DNA transfection cooperating with a cationic lipid bearing ethylenediamine. Biochim. Biophys. Acta.

[23] Guo X., Huang L. (2011). Recent advances in nonviral vectors for gene delivery. Acc. Chem. Res..

[24] Falsini S., Ciani L., Ristori S., Fortunato A., Arcangeli A. (2013). Advances in lipid-based platforms for RNAi therapeutics. J. Med. Chem..

[25] Mével M., Kamaly N., Carmona S., Oliver M.H., Jorgensen M.R., Crowther C., Salazar F.H., Marion P.L., Fujino M., Natori Y. (2010). DODAG; a versatile new cationic lipid that mediates efficient delivery of pDNA and siRNA. J. Control. Release.

[26] Li Y., Zhu Y., Xia K., Sheng R., Jia L., Hou X., Xu Y., Cao A. (2009). Dendritic poly(l-lysine)-*b*-poly(l-lactide)-*b*-dendritic poly(l-lysine) amphiphilic gene delivery vectors: Roles of PLL dendritic generation and enhanced transgene efficacies via termini modification. Biomacromolecules.

[27] Zheng Y., Liu X., Samoshina N.M., Samoshin V.V., Franz A.H., Guo X. (2015). *trans*-2-Aminocyclohexanol-based amphiphiles as highly efficient helper lipids for gene delivery by lipoplexes. Biochim. Biophys. Acta.

[28] Chen C.J., Wang J.C., Zhao E.Y., Gao L.Y., Feng Q., Liu X.Y., Zhao Z.X., Ma X.F., Hou W.J., Zhang L.R. (2013). Self-assembly cationic nanoparticles based on cholesterol-grafted bioreducible poly(amidoamine) for siRNA delivery. Biomaterials.

[29] Jones C.H., Chen C.K., Jiang M., Fang L., Cheng C., Pfeifer B.A. (2013). Synthesis of cationic polylactides with tunable charge densities as nanocarriers for effective gene delivery. Mol. Pharm..

[30] Fàbregas A., Sánchez-Hernández N., Ticó J.R., García-Montoya E., Pérez-Lozano P., Suñé-Negre J.M., Hernández-Munain C., Suñé C., Miñarro M. (2014). A new optimized formulation of cationic solid lipid nanoparticles intended for gene delivery: Development, characterization and DNA binding efficiency of TCERG1 expression plasmid. Int. J. Pharm..

[31] Rehman Z.U., Zuhorn I.S., Hoekstra D. (2013). How cationic lipids transfer nucleic acids into cells and across cellular membranes: Recent advances. J. Control. Release.

[32] Zhang Q.-F., Yi W.-J., Wang B., Zhang J., Ren L., Chen Q.-M., Guo L., Yu X.Q. (2013). Linear polycations by ring-opening polymerization as non-viral gene delivery vectors. Biomaterials.

[33] Qin B., Chen Z., Jin W., Cheng K. (2013). Development of cholesteryl peptide micelles for siRNA delivery. J. Control. Release.

[34] Ma B., Zhang S., Jiang H., Zhao B., Lv H. (2007). Lipoplex morphologies and their influences on transfection efficiency in gene delivery. J. Control. Release.

[35] Yu F., Yang Y., Wang A., Hu B., Luo X., Sheng R., Dong Y., Fan W. (2015). Quantitative and highly selective sensing of sodium houttuyfonate via long-aliphatic chains hydrophobic assembly and aggregation-induced emission. New J. Chem..

[36] Zuhorn I.S., Bakowsky U., Polushkin E., Visser W.H., Stuart M.C.A., Engberts J.B.F.N., Hoekstra D. (2005). Nonbilayer phase of lipoplex-membrane mixture determines endosomal escape of genetic cargo and transfection efficiency. Mol. Therapy.

[37] Sheng R., An F., Wang Z., Li M., Cao A. (2015). Assembly of plasmid DNA with pyrene-amines cationic amphiphiles into nanoparticles and their visible lysosome localization. RSC Adv..

[38] Sheng R., Xia K., Chen J., Xu Y., Cao A. (2013). Terminal modification on mPEG-dendritic poly-(*l*)-lysine cationic diblock copolymer for efficient gene delivery. J. Biomater. Sci. Polym. Ed..

[39] Vighi E., Montanari M., Hanuskova M., Iannuccelli V., Coppi G., Leo E. (2013). Design flexibility influencing the *in vitro* behavior of cationic SLN as a nonviral gene vector. Int. J. Pharm..

[40] Swaney W., Sorgi F., Bahnson A., Barranger J. (1997). The effect of cationic liposome pretreatment and centrifugation on retrovirus-mediated gene transfer. Gene Therapy.

[41] Shi B., Zhang H., Shen Z., Bi J., Dai S. (2013). Developing a chitosan supported imidazole Schiff-base for high-efficiency gene delivery. Polym. Chem..

[42] Perez A.P., Cosaka M.L., Romero E.L., Morilla M.J. (2011). Uptake and intracellular traffic of siRNA dendriplexes in glioblastoma cells and macrophages. Int. J. Nanomed..

[43] Li H., Luo T., Sheng R., Sun J., Wang Z., Cao A. (2013). Endoplasmic reticulum localization of poly(ω-aminohexyl methacrylamide)s conjugated with (l-)-arginines in plasmid DNA delivery. Biomaterials.

[44] Mével M., Haudebourg T., Colombani T., Peuziat P., Dallet L., Chatin B., Lambert O., Berchel M., Montier T., Jaffrès P.A. (2015). Important role of phosphoramido linkage in imidazole-based dioleyl helper lipids for liposome stability and primary cell transfection. J. Gene Med..

[45] Koynova R., Tenchov B., MacDonald R.C. (2015). Nonlamellar phases in cationic phospholipids, relevance to drug and gene delivery. ACS Biomater. Sci. Eng..

[46] Khalil I.A., Kogure K., Akita H., Harashima H. (2006). Uptake pathways and subsequent intracellular trafficking in nonviral gene delivery. Pharm. Rev..

[47] Kou L., Sun J., Zhai Y., He Z. (2013). The endocytosis and intracellular fate of nanomedicines: Implication for rational design. Asia J. Pharm. Sci..

[48] Chu Z., Miu K., Lung P., Zhang S., Zhao S., Chang H.-C., Lin G., Li Q. (2015). Rapid endosomal escape of prickly nanodiamonds: Implications for gene delivery. Sci. Rep..

[49] Yang N., Caratti G., Ince L.M., Poolman T.M., Trebble P.J., Holt C.M., Ray D.W., Matthews L.C. (2014). Serum cholesterol selectively regulates glucocorticoid sensitivity through activation of JNK. J. Endocrinol..

[50] Zeng X., Morgenstern R., Nyström A.M. (2014). Nanoparticle-directed sub-cellular localization of doxorubicin and the sensitization breast cancer cells by circumventing GST-Mediated drug resistance. Biomaterials.

[51] Xu X., Ho W., Zhang X., Bertrand N., Farokhzad O. (2015). Cancer nanomedicine: From targeted delivery to combination therapy. Trend. Mol. Med..

