# Supplementary material for: Cationic Nanoparticles Assembled from Natural-Based Steroid Lipid for Improved Intracellular Transport of siRNA and pDNA
Source: Nanomaterials (Basel). 2016 Apr 13;6(4):69. doi: 10.3390/nano6040069 (PMC5302561; doi:10.3390/nano6040069)
Supplement: Supplementary file 1 [file nanomaterials-06-00069-s001.pdf]

Supplementary Materials: Cationic Nanoparticles Assembled from Natural-Based Steroid Lipid for Improved Intracellular Transport of siRNA and pDNA

Ruilong Sheng, Xiaoqing Zhuang, Zhao Wang, Amin Cao, Kaili Lin and Julian X. X. Zhu

S1. Synthesis and Characterization of the Cationic Lipid Diosarg

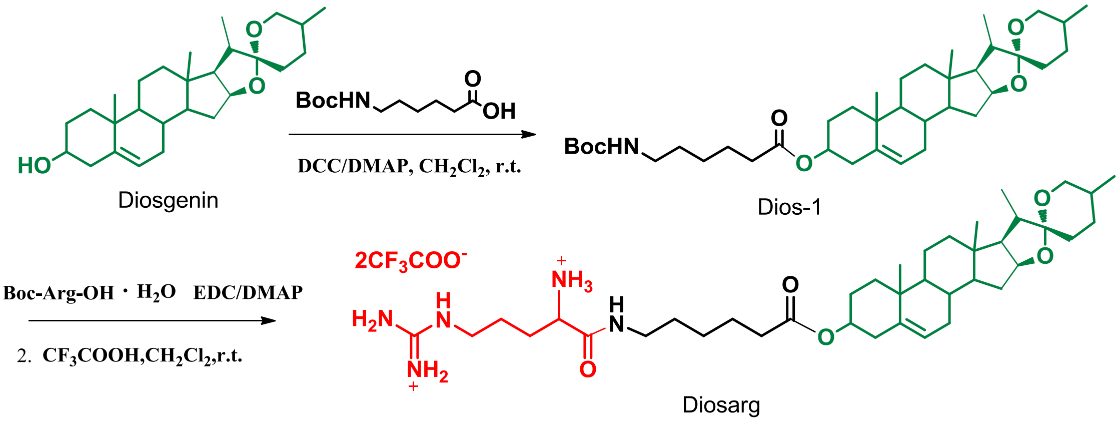

Scheme S1. The synthesis routes of the cationic lipid Diosarg. (DCC: N,N'-dicyclohexylcarbodiimide, EDC: 1-Ethyl-3-(3-dimethylaminopropyl)carbodiimide, DMAP: 4-Dimethylaminopyridine)

The synthesis procedures of the cationic lipid Diosarg:

Step 1: In a 100-mL flask were added diosgenin (414 mg, 1.0 mmol), Boc-N-caproic acid (231 mg, 1.5 mmol) and N,N'-dicyclohexylcarbodiimide (DCC)/4-Dimethylaminopyridine (DMAP) (300 mg/100 mg) dissolved in 50 mL of dichloromethane, and the mixture was stirred at ambient temperature for 24 h. Then, the dicyclohexylurea (DCU) solids were removed by filtration; the residual solution was further concentrated and then purified by flash column chromatography (EtOAC/hexane = 1/3 *v*/*v*) to achieve Dios-1 as a white solid (isolated yield: 66.1%).

Step 2: In a 50-mL flask, the Dios-1 (320 mg, 0.5 mmol) was dissolved in the mixture solution of CH_2Cl_2/CF_3COOH (20 mL/5 mL) and stirred at ambient temperature for 2 h. Then, the solvent was removed under reduced pressure, and then Boc-protected arginine Boc-Arg·HCl·H_2O (250 mg, 0.75 mol), 1-Ethyl-3-(3-dimethylaminopropyl)carbodiimide (EDC)/DMAP (300 mg/100 mg), which was dissolved in the mixture solution of CH_2Cl_2/triethylamine (50 mL/5 mL), were added; then, the mixture was stirred at ambient temperature for 24 h and then washed with distilled water (3 × 100 mL), then concentrated under reduced pressure and purified by flash column chromatography (eluent: EtOAC/MeOH = 10/1 *v*/*v*) to get the Boc-protected Diosarg lipid precursor as a yellowish solid. Finally, the Boc groups were removed in CH_2Cl_2/CF_3COOH (5 mL/5 mL) after a 3-h reaction at room temperature, then the solvent was removed and precipitated with diethyl ether. The final product, cationic lipid Diosarg, was obtained as a white solid (isolated yield: 59.6%).

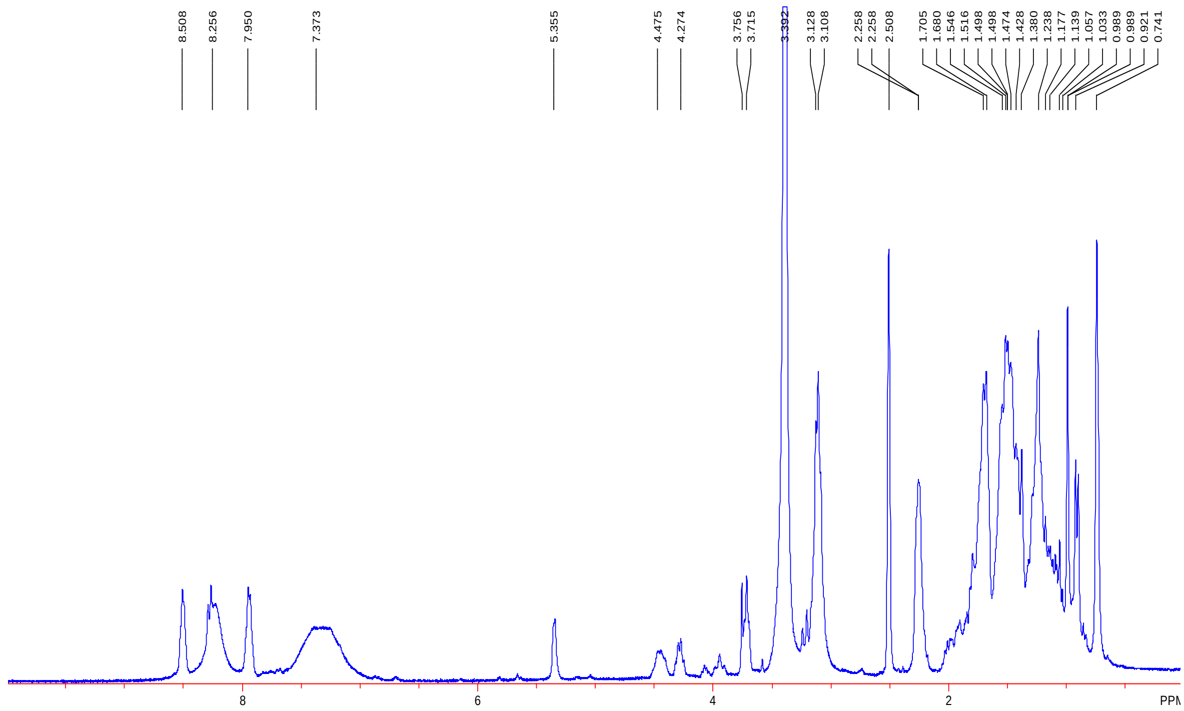

Figure S1. ^{1}H NMR spectra of the cationic lipid Diosarg.

Characterization data of the cationic lipid Diosarg:

^{1}H NMR (d_4-MeOD, 300 Hz): δ 7.70–8.50 (guanidyl-H, arginine), 7.0–7.60 (H, NH_3^+), 5.40 (1H, –C$\underline{H}$=C, diosgenin), 4.51 (1H, COO–C$\underline{H}$–), 4.18 (2H, OCO–C$\underline{H}_2$, diosgenin), 3.11 (2H, Boc-NH–C$\underline{H}_2$), 2.30 (2H, –C$\underline{H}_2$COO–), 1.42 (9H, Boc), 2.23–0.61 (45H, diosgenin).

^{13}C NMR (d_4-MeOD, 75 Hz): δ 172.7, 168.4, 159.2, 157.3, 139.7, 119.1, 116.0, 108.7, 80.5, 73.9, 66.4, 65.8, 56.2, 52.2, 49.5, 41.5, 40.5, 40.1, 39.8, 39.2, 38.4, 36.8, 36.6, 34.8, 34.1, 32.0, 31.3, 31.1, 28.9, 28.7, 25.9, 25.0, 24.2, 19.6, 18.7, 17.5, 15.1.

ESI-MS: [M^+] = 684.5 (*m*/*z*), calculated: 684.5

S2. Hydrodynamic Average Particle Sizes of the Dios-DOPE (2:1, 1:1, 1:2) NPs in Distilled Water at Room Temperature Measured by the Dynamic Light Scattering Instrument

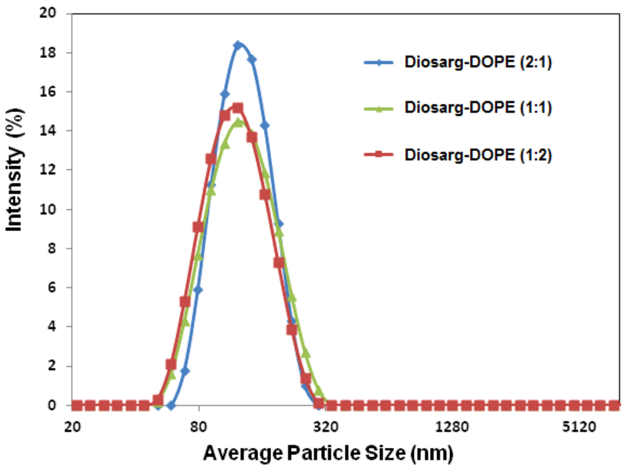

Figure S2. Hydrodynamic average particle sizes of the Diosarg-1,2-dioleoyl-sn-glycero-3-phosphor-ethanolamine (DOPE) (2:1, 1:1, 1:2) NPs in distilled water at room temperature measured by the dynamic light scattering instrument.

S3. Agarose-Gel pDNA Retardation Assay of the pDNA Binding Affinity for the Diosarg Lipid and Diosarg-DOPE NPs

Experimental procedures of the agarose-gel pDNA retardation assay:

Before the assay, the cationic lipid samples (Diosarg and Diosarg-DOPE NPs) were preliminarily dissolved in pure water to prepare the solution with the total Diosarg concentration of 1.7×10^{-3} M (same Diosarg concentration for all of the Diosarg and Diosarg-DOPE NPs); the Diosarg/pDNA and Diosarg-DOPE NPs/pDNA complexes were prepared by mixing pDNA (1 μg) with a predetermined amount of the cationic lipid sample stock solutions under a pre-set ± charge ratio in 50 μL PBS (0.01 M, pH = 7.4) buffer solution. After incubation at 37 °C for 20 min, the Diosarg/pDNA and Diosarg-DOPE NPs/pDNA complex solution were loaded onto a 1% agarose gel containing 0.5 μg/mL ethidium bromide dye (EB). Then, the gel electrophoresis was conducted in 1 × TAE running buffer under 100 mV for 30 minutes, and the pDNA migration bands were thus observed and recorded on a UVP benchtop 2UV transilluminator system.

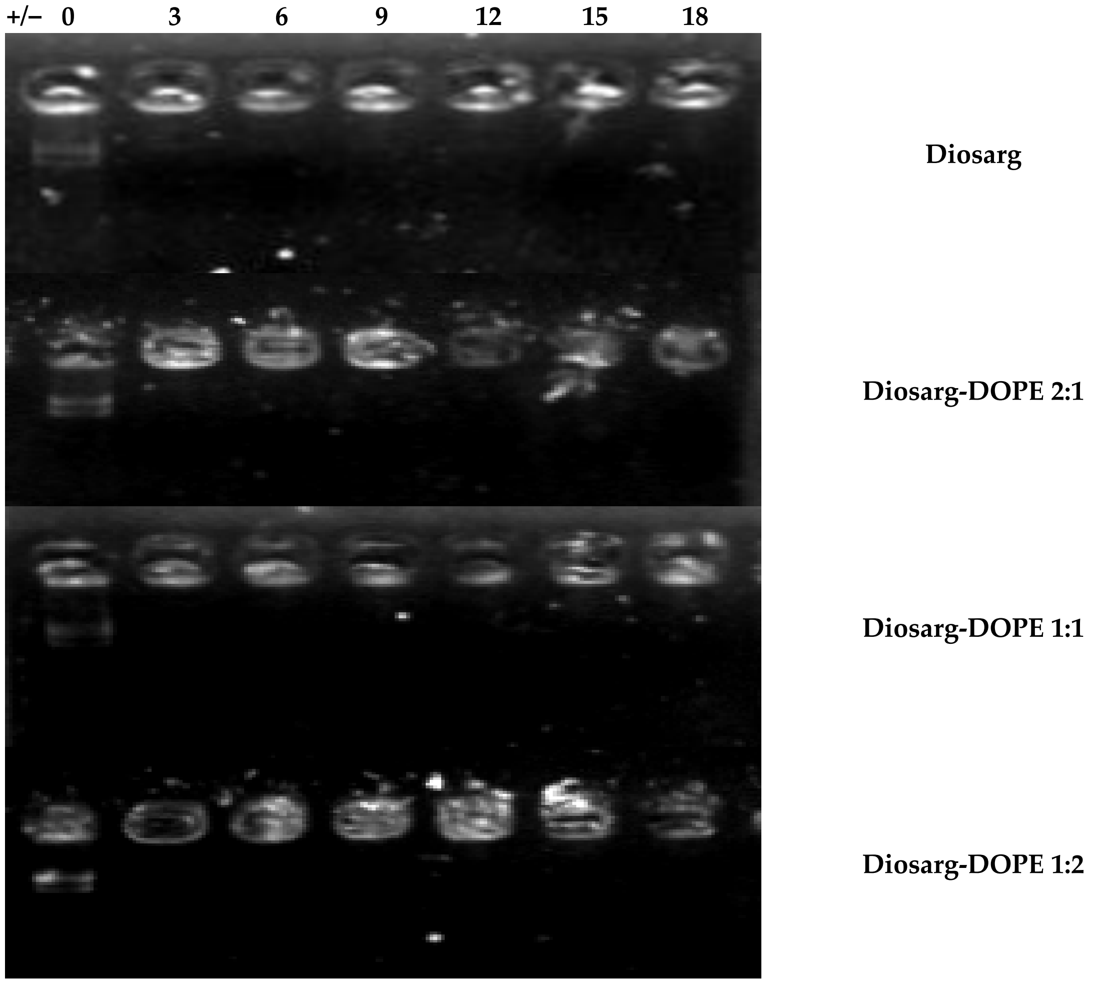

Figure S3. Agarose-gel pDNA retardation assay of the pDNA binding affinity for the Diosarg lipid and Diosarg-DOPE NPs; in each well, pDNA (1 μg) mixed with the Diosarg lipids and Diosarg-DOPE (2:1, 1:1, 1:2) NPs under various ± charge ratios (0–12), respectively.

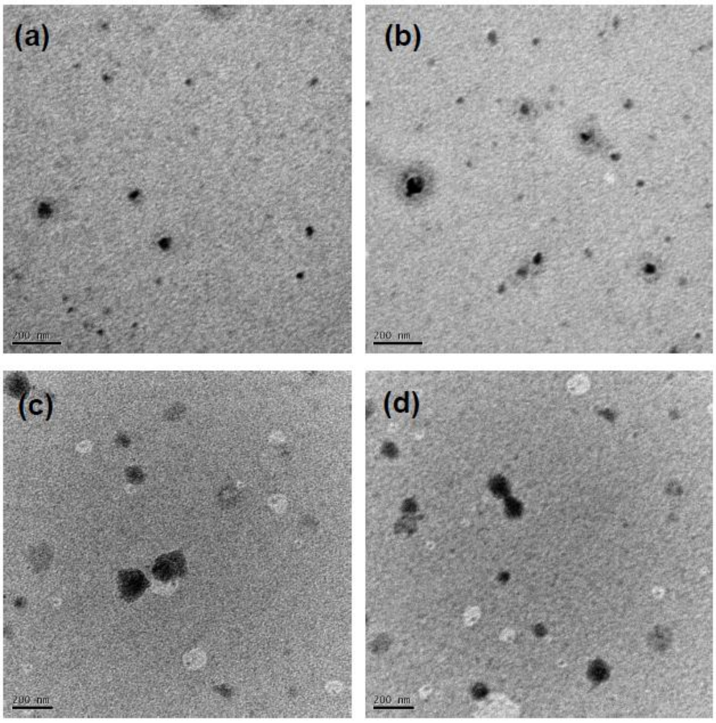

Figure S4. The morphology of the Diosarg-DOPE (2:1)/siRNA (**a**), Diosarg-DOPE (1:1)/siRNA (**b**), Diosarg-DOPE (2:1)/pDNA (**c**) and Diosarg-DOPE (1:1)/pDNA (**d**) complexes under a ± charge ratio of 15 was observed by transmission electron microscopy (TEM).

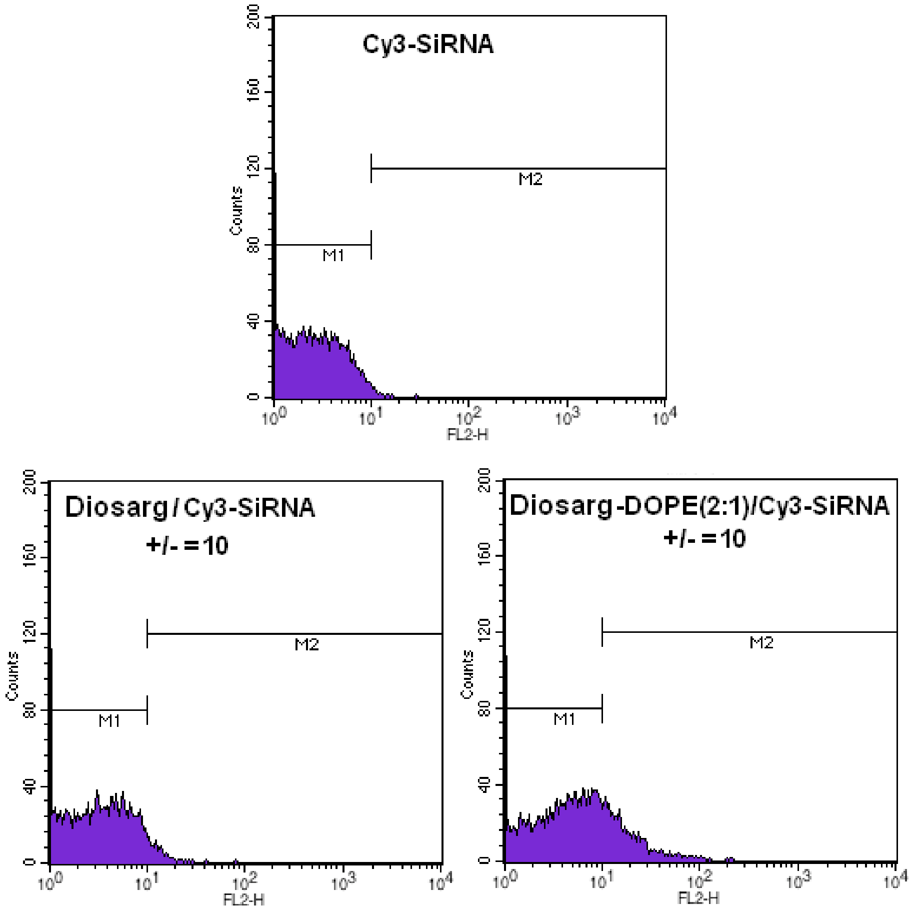

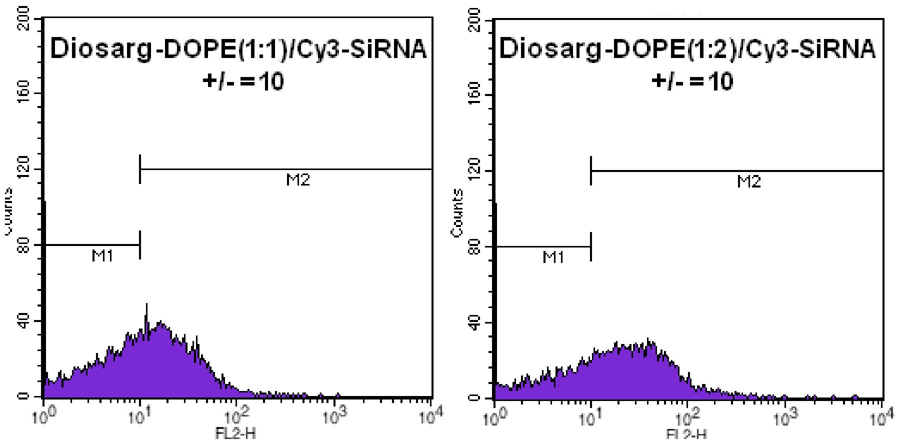

Figure S5. Intracellular uptake of the Diosarg lipid and Diosarg-DOPE (2:1, 1:1, 1:2) NPs/Cy3-siRNA complexes (± = 10) in H1299 cells measured by flow cytometry (10,000 cells for each sample).

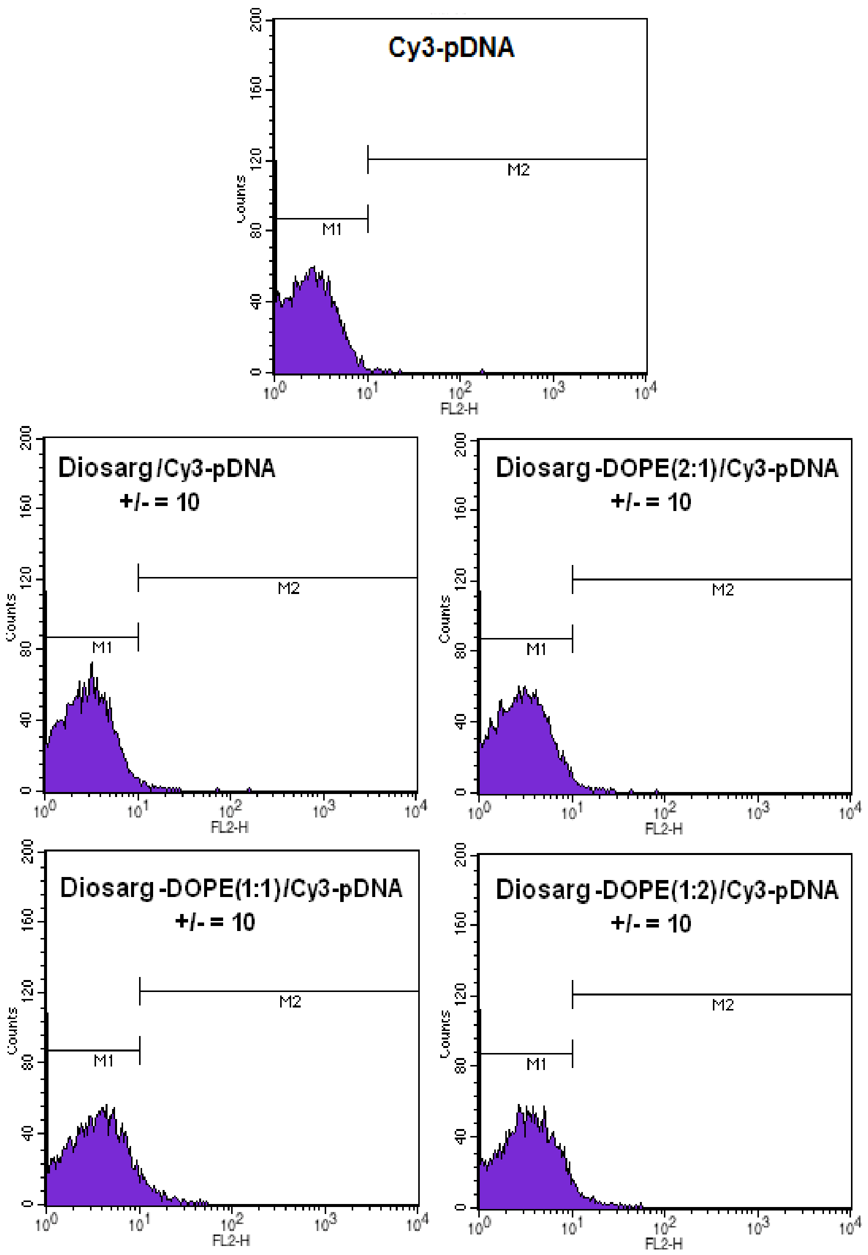

Figure S6. Intracellular uptake of the Diosarg lipid and Diosarg-DOPE (2:1, 1:1, 1:2) NPs/Cy3-pDNA complexes (± = 10) in H1299 cells measured by flow cytometry (10,000 cells for each sample).

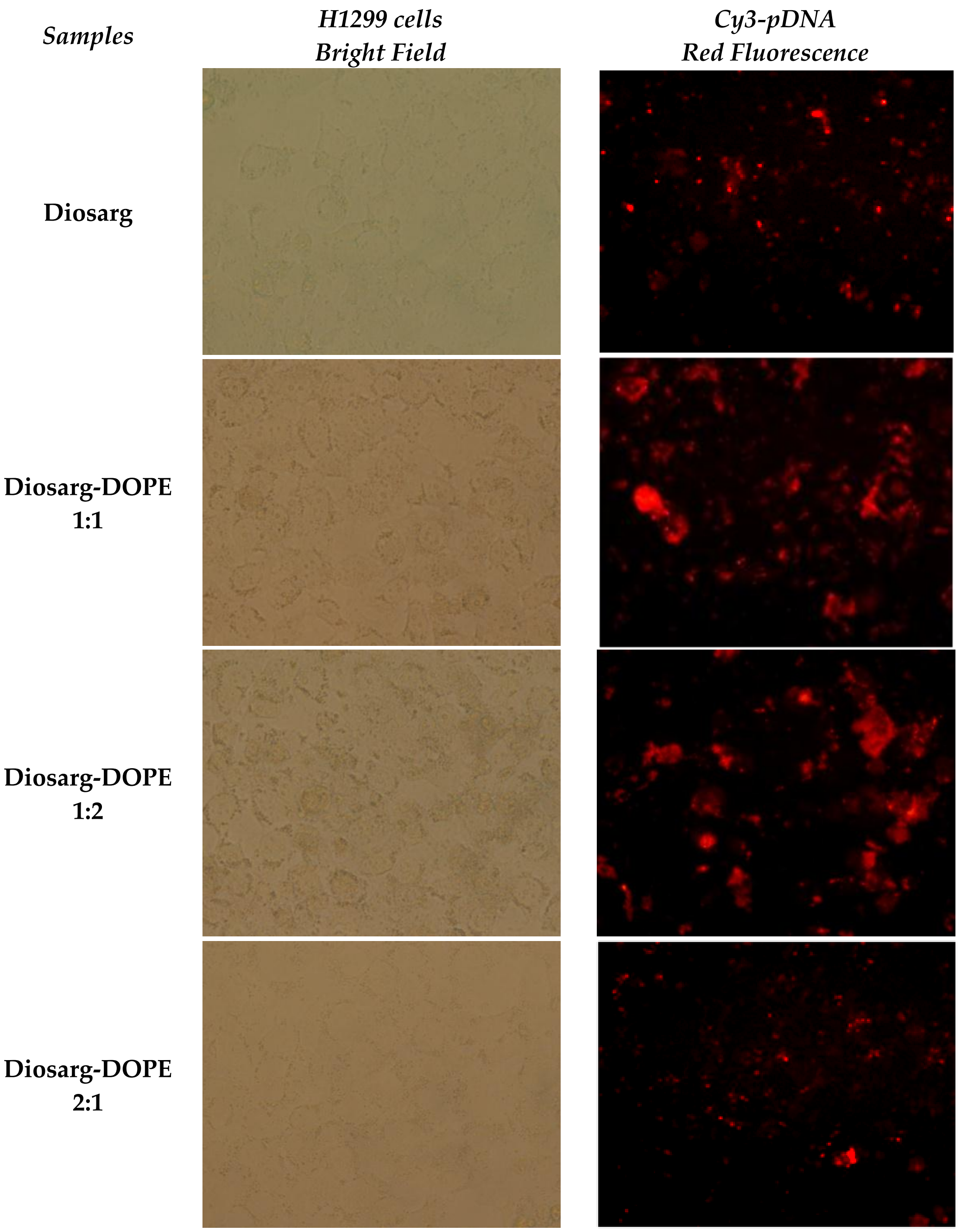

Figure S7. Fluorescence imaging of H1299 cells after six hours of incubation of the Diosarg/Cy3-pDNA and Diosarg-DOPE (2:1, 1:1, 1:2) NPs/Cy3-pDNA under a ± ratio of 15 indicated that the co-assembly of DOPE with the cationic Diosarg lipid could enhance the uptake of Cy3-pDNA.

© 2016 by the authors; licensee MDPI, Basel, Switzerland. This article is an open access article distributed under the terms and conditions of the Creative Commons by Attribution (CC-BY) license (http://creativecommons.org/licenses/by/4.0/).
